# Stated preferences for anti-malarial drug characteristics in Zomba, a malaria endemic area of Malawi

**DOI:** 10.1186/1475-2875-13-259

**Published:** 2014-07-08

**Authors:** Antonieta Medina-Lara, Ruben E Mujica-Mota, Esthery D Kunkwenzu, David G Lalloo

**Affiliations:** 1 Health Economics Group, University of Exeter Medical School, University of Exeter, Veysey Building, Salmon Pool Lane, Exeter EX2 4SG, UK; 2 Institute of Health Research, University of Exeter Medical School, University of Exeter, Vesey Building, Salmon Pool Lane, Exeter EX2 5GU, UK; 3 University of Malawi, Chancellor College, Box 280, Zomba, Malawi; 4 Liverpool School of Tropical Medicine, Pembroke Place, Liverpool L3 5QA, UK

**Keywords:** Stated preference experiments, Anti-malarials, Malaria, Africa

## Abstract

**Background:**

The evidence on determinants of individuals’ choices for anti-malarial drug treatments is scarce. This study sought to measure the strength of preference for adult antimalarial drug treatment attributes of heads of urban, rural and peri-urban households in a resource-limited malaria-endemic area of sub-Saharan Africa.

**Methods:**

Discrete choice experiments were conducted with 508 heads of household interviewed face-to-face for a household population survey of health-seeking behavior in Zomba District, Malawi. The interviews were held in Chichewa and the choice experiment questions were presented with cartoon aids. The anti-malarial drug attributes included in the stated preference experiment were: speed of fever resolution, side effects (pruritus) risk, protection (duration of prophylactic effect), price, duration of treatment course and recommendation by a health professional. Sixteen treatment profiles from a fractional factorial design by orthogonal array were paired into choice scenarios, and scenarios were randomly assigned to participants so that each participant was presented with a series of eight pairwise choice scenarios. Respondents had the option to state indifference between the two profiles or decline to choose. Data were analysed in a mixed logit model, with normally distributed coefficients for all six attributes.

**Results:**

The sex ratio was balanced in urban areas, whereas 63% of participants in rural areas were male. The proportion of individuals with no education was considerably higher in the rural group (25%) than in the urban (5%) and peri-urban (6%) groups. All attributes investigated had the expected influence, and traded-off in most respondents’ choices. There were heterogeneous effects of price, pruritus risk, treatment recommendation by a professional, and duration of prophylaxis across respondents, only partly explained by their differences in education, household per capita expenditure, sex and age. Individuals´ demand elasticity (simulated median, inter-quartile range) was highest (most responsive) to speed of symptom resolution (0.88, 0.80-0.89) and pruritus risk (0.25, 0.08-0.62).

**Conclusions:**

Most adult antimalarial users are willing to use treatments without recommendation from health professional, and may be influenced by price. Future studies should investigate the magnitude of differences in price and treatment attribute sensitivity between adult anti-malarial drug users in rural, peri-urban and urban areas in order to determine optimal price subsidies.

## Background

In sub-Saharan Africa, anti-malarial drug treatment may be accessed through the informal and formal sectors. Self-medication through purchases from kiosks or pharmacies is common, providing over the counter access to a range of treatments, including drugs that are not part of the government’s national anti-malarial programme and prescription-only drugs reserved for second-line treatment [1,2]. Despite the fact that there is growing evidence about treatment-seeking behaviour patterns and strategies used by households to cope with symptoms resembling malaria in endemic countries [3-11], there is limited evidence on the factors that determine the choices made by individuals who purchase anti-malarial medication from shops and pharmacies.

In western Uganda, a study examined the acceptability of drug presentation characteristics by asking women aged 15–49 to state a preference for pre-packed or conventional, loose tablet dispensing of anti-malarial drugs for children at government health facilities, including the reasons for their preference, their willingness to pay for pre-packaging, and their actions in the hypothetical event that the pre-packed dose for the required child age was not available [12]. However, no study appears to have considered the role of the attributes of the anti-malarial drugs themselves on treatment choice. Identification of factors that are seen as desirable by consumers of anti-malarials would help to inform the design of interventions to improve the management of cases presenting with malaria-like symptoms in the community and would also allow policy-makers to design more effective strategies for the implementation of anti-malarial treatment policy in the region. Such information is particularly important at a time when artemisinin combination therapy (ACT) is now used as first-line therapy throughout much of Africa and is increasingly available in the informal sector across Africa.

Stated preference methods provide a rigorous, systematic framework for inferring such factors, by examining responses to hypothetical choices between two or more prototype drugs defined in terms of a set of characteristics believed to represent attributes relevant to real individual purchasing decisions. Since hypothetical drug options presented to any given individual are generated randomly from the range of possible values that each and every attribute may take, associations of drug choices and characteristics may be given a causal interpretation. Moreover, by seeking to replicate a real life decision situation, this technique provides an alternative approach to explicitly eliciting answers from respondents about desirable attributes and their importance. In health, stated preference methods, sometimes misleadingly referred to as conjoint analyses, have been used to explore issues ranging from the evaluation of prescription decisions [13], the introduction of chicken pox vaccination [14], to patient preferences for managing asthma [15].

This study aims to identify individuals’ strength of preference for combinations of anti-malarial drug attributes in the Zomba District of Malawi. Attributes investigated relate to the clinical effects of the drug, cost of acquiring the drug in the market, the duration of treatment and the status of the drug in the prescribing recommendations of health professionals. This study does not consider issues of access or preferred source of drug, which although pertinent to choice in real life, need to be studied separately.

## Methods

The factors chosen to be evaluated in our stated preference study were identified by a prior qualitative study conducted in three sites in rural and peri-urban areas in Zomba. Focus group discussions (FGDs) were held with adults to elicit their views on the most important anti-malarial drug characteristics and their ranking. A total of 13 FGDs, each with 5–10 participants, were conducted; six FGDs included only men, six were held exclusively with women and one had a mixed gender composition for verifying and validating emerging issues and themes. The total number of FGDs was determined by the attainment of saturation (i.e., when new groups stopped adding new information to the discussion).

The qualitative study identified and ranked the following as the most important attributes of anti-malarial drugs: speed of fever resolution, side effects, protection (prophylaxis) and price. Duration of treatment course and recommendation by a health professional were also included, since they were implicitly referred to as being important by respondents in the qualitative study.

Once the attributes were identified, each of the characteristics was assigned realistic levels i.e., time to fever resolution, 24 hrs. vs. 12 hrs.; side effects defined as the risk of rash (pruritus), 1 vs. 3 in 10 individuals; protection from re-infection (prophylactic effect), 10 days vs. 50 days; price paid, 500 Kwachas (US$4.62, high; $1 = Malawian Kwacha108.84), 200 Kwachas (US$1.85, medium), and 50 Kwachas (US$0.46, low); duration of treatment course, 1 vs. 3 days; and whether recommended by a health professional (yes or no). These values were intended to encompass the characteristics of sulfadoxine-pyrimethamine (SP) and ACT, respectively, the former and new official first-line treatments in Malawi. The terms ‘price’ and ‘cost’ are used interchangeably in what follows (since the price of antimalarials obtained from shops and pharmacies is paid by the user), to facilitate continuity in exposition between statistical models, which differ in how the price attribute is included in the model (see Data analysis).

The specification of experimental profiles was a fractional factorial design determined by an orthogonal array, with a quasi-random selection of attribute levels restricted to the minimum number of profiles that allows estimation of the attribute (main) effects. The resulting 16 profiles were used to form all possible pairs of distinct profiles, 120, each pair representing an unlabelled choice question. Eight different choice questions were randomly allocated and presented to each household head in a community survey of health-seeking behaviour for treating fever in adults and children in the Zomba District of Malawi. The methodological details of the survey have been reported elsewhere [16]. Briefly, households were selected by two-stage cluster sampling with stratification (by area; rural and urban vs. peri-urban) from all the traditional authority units in Zomba. Participants living in urban (all townships, town planning areas and district centres), peri-urban (settlements within 20 km of urban boundaries) and rural areas (others) were face-to-face interviewed in the local language, Chichewa, by trained fieldworkers. In each choice question, the respondent had the option to state one of the two profiles as their preferred treatment for their own malaria or that they had no preference between the two options or that they would refuse choosing any of the two options. Additional information on the household head’s age, sex, education, occupation, experience of using an anti-malarial drug, anti-malarial drug experienced, sleeping under a bednet the previous night, and household per capita monthly expenditure, was used to analyse patterns of refusal to make a selection and heterogeneity in attribute effects.

This design corresponds to a ratio of 120 options in different combination sets to 14 degrees of freedom, which provides adequate power for estimating all main effects [17]. In cases where the random allocation yielded a choice question involving the same profile in both options, the random draw was repeated until the question presented the respondent with a choice between two different profiles. The final design had a block diagonal covariance matrix and it was 57% D-efficient [18].

Cartoon aids (Additional file 1) were used to present the experimental choice questions to respondents. The format was pilot tested in a sample of participants (n = 16). The use of cartoons was found to facilitate the cognitive task demanded by the experiment, in particular the communication of magnitudes of pruritus risk and treatment duration. The pilot questionnaire took between 10–15 minutes to complete and no difficulties with understanding questions or providing responses were reported by the participants. In particular, the tools were found to yield valid answers among illiterate respondents, the majority of whom were found in rural areas.

Data collection forms were standardized and double entered into SPSS databases. Only study numbers were used as personal identifiers. Data records were treated confidentially and only available to the staff directly concerned with this research. Ethical approval was obtained from the University of Zomba in Malawi and the Research Ethics Committee of the Liverpool School of Tropical Medicine. No monetary compensation was given to study participants.

### Data analysis

Responses to choice questions were analysed according to a model of random utility where, in a choice scenario involving two options, an option, say ‘Option 1’, would be selected according to the following principle:

U(Option 1) > U(Option 2)

where U, the utility of an option, is a function of its attributes. Utility is represented as a latent linear function; that is:

(1) $$\begin{array}{ll} U\left({\mathrm{Option}}_{j}\right)= & {\mathrm{\alpha C}}_{j}+{\mathrm{\nu F}}_{j}+\gamma \;R_{j}+\delta \;P_{j}+{\mathrm{\zeta D}}_{j}\\ & +{\mathrm{\eta H}}_{j}+\epsilon_{j}, \end{array}$$

where C is the drug price, F is time to fever resolution, R is pruritus (rash) risk, P is the duration of protection from reinfection, D is the treatment duration, and H indicates whether the drug is recommended by a health professional; α, ν, γ, δ, ζ, η are the parameters (effects) to be estimated and ϵ is the random part of the model. The effect on the drug option’s utility of a $1 increase in its price, α, is equal to the negative of the marginal utility of income under the assumption of constant marginal utility, and may thus serve as reference to value the other effects. For example, the ratio ζ/α, gives the monetary rate or price reduction an individual is willing to accept as compensation for one extra day of drug therapy; i.e. the marginal willingness to pay for one day less of treatment (since both parameters are expected to be negative). The selection of Option 1, say, is interpreted here as:

$$\begin{array}{ll} {\mathrm{\alpha C}}_{1} & +{\mathrm{\nu F}}_{1}+{\mathrm{\gamma R}}_{1}+\delta \;P_{1}+{\mathrm{\zeta D}}_{1}+{\mathrm{\eta H}}_{1}+\epsilon_{1}>{\mathrm{\alpha C}}_{2}\\ & +{\mathrm{\nu F}}_{2}+{\mathrm{\gamma R}}_{2}+\delta \;P_{2}+{\mathrm{\zeta D}}_{2}+{\mathrm{\eta H}}_{2}+\epsilon_{2} \end{array}$$

which may be expressed as:

$$\beta 'X_{1}+\epsilon_{1}>\beta 'X_{2}+\epsilon_{2}$$

or equivalently:

(2) $$\mathrm{\alpha \Delta C}+\mathrm{\nu \Delta F}+\mathrm{\gamma \Delta R}+\mathrm{\delta \Delta P}+\mathrm{\zeta \Delta D}+\mathrm{\eta \Delta H}>\epsilon_{2}-\epsilon_{1}$$

where variables with the suffix 1 refer to the characteristics of Option 1, while variables with suffix 2 refer to the other option, and β’X is the shorthand expression of the linear combination of attribute and coefficient products (β’ stands for the transposed vector of parameters and X for the vector of attributes) and Δ denotes the value of an attribute of option 1 minus the corresponding value for option 2. Thus, an option with a lower cost than the alternative and equal values for all other attributes and distribution of error term, will have a higher probability of being chosen as long as α is negative. The probability will depend on the specific value of α and the probability distribution of the random error terms ϵ1 and ϵ2. The most common distributions imposed on these terms include the logistic (logit) and normal (probit) distribution, since they involve simple probability functions (the probit is more complex in the case of more than two options). In general, the choice between the two makes no significant difference [19].

The (random effects) logit (i.e. mixed logit) form was chosen since it combines the convenience of the logit form with the simplicity of modelling the effect of unobserved individual heterogeneity in the attribute effects in answers to repeated choice questions by the same individual as random normal coefficients. Thus, a random error varying across individuals but remaining constant within choices by the same individual was specified for each attribute’s coefficient, assuming independence between errors of different coefficients. In addition heteroscedasticity across location of residence was allowed for by two scale parameters: one associated with living in an urban area and another for observations from peri-urban areas.

The model was estimated using simulated maximum likelihood, by modifying the likelihood and gradient functions of the GAUSS (Mathematical and Statistical System™) programme mixed logit estimation by maximum simulated likelihood by Kenneth Train [20], with a modification to allow for heteroscedasticity using different variance scale parameters for rural, urban and peri-urban observations. The likelihood (L) of each choice by an individual in the model was:

(3) $$\begin{array}{ll} L= & y*\frac{\exp{\left(\beta ’X1\right)}^{s}}{\left[\sum_{i=1}^{2}\exp{\left(\beta ’\mathrm{Xi}\right)}^{s}\right]}+\left(1-y\right)\\ & *\frac{\exp{\left(\beta ’X2\right)}^{s}}{\left[\sum_{i=1}^{2}\exp{\left(\beta ’\mathrm{Xi}\right)}^{s}\right]} \end{array}$$

Where y is the choice indicator taking the value of 1 if option 1 is selected and 0 otherwise; β’x1 and β’x2 are the vectors of attributes and coefficients for the two options in Equation 2, and the βs are random normal parameters; the term s is a variance scale parameter ≡ mu + 1-u, taking the value of 1 if the respondent lives in a rural area (u=0) or m, if resident of an urban settlement (u=1). The parameter m is simply the ratio of the variance of responses from persons in rural areas to the variance of responses from urban residents: a value of 1 implies equal urban and rural variances whilst higher values signal higher rural variance. Two variance scale parameters (relative to rural variance) were estimated, one for urban and another for peri-urban residents. The effect of a (marginal) change in the value of an attribute is a (nonlinear) function of the initial covariate (attribute) values, the βs and the value of m; this allows for the possibility of the same change having a different effect on the probability of choice in the three types of population settlement; the direction of effect is given by the sign of the coefficient of the varying attribute. The product of likelihood terms as Equation 3 for all choices across all individuals is the likelihood function; the estimated parameters are the value of the βs (and their standard deviations) and the scale variance terms at which the function achieves its maximum value. Weights were used in estimation to reflect the survey design [16] and to obtain representative attribute coefficient estimates for the head of household population in the district.

The working hypothesis is thus that the separate effects of a higher cost, greater risk of side effects (i.e., rash), longer time to fever resolution, and longer treatment course duration are negative, so that higher values for these attributes are considered to imply less attractive drug options, whereas recommendation of the drug in question by a health professional and a longer duration of prophylactic effect are expected to make an anti-malarial drug treatment option more attractive to users. Results are presented for the attribute coefficients in the linear index (β in Equation 2) and, for attributes other than health professional recommendation, as the ratio of relative change in the probability of selection to the proportional change in the respective attribute level (elasticity); for the health professional recommendation attribute, the odds ratio was used instead of the elasticity, since this was not defined.

## Results

The stated preference questionnaire was presented to the 508 household heads included in the community survey. From these, 55 household heads (13%) had one or more choice data missing from their records, resulting in 164(4.04%) unanswered choice questions, with one participant declining to answer the questionnaire. Responses of all individuals were included in the analysis.

Table 1 shows descriptive statistics about the participants, including socio-economic characteristics, malaria prevention behaviour, experience with anti-malarial treatment and the proportion of questions where individuals declined to select any of the two options presented. The sex ratio was equal in urban and peri-urban areas, whereas 63% of participants in rural areas were male. The proportion of individuals with no education is considerably higher in the rural group (25% vs. 6.2%, in peri-urban, and 4.7%, in urban), as is their lack of access to essential means of information (i.e. no radio at home; 31% vs. 11% and 2%). The experience of anti-malarial drug consumption appears to be more varied in urban areas than the rest, with the proportion in rural areas not having a preferred anti-malarial being twice that in urban and peri-urban areas. No-one in rural or peri-urban areas chose halofantrine or mefloquine, drugs that were not part of the official treatment policy, as their preferred treatment, although this was the choice for 9.4% of urban respondents.

**Table 1:** Choice data and participant characteristics

**Variables**	**Rural**	**Peri-Urban**	**Urban**
N of participants	364	80	64
N of scenarios	2911	640	512
N of scenarios where no drug profile was chosen (%)	129 (4.4)	23 (3.6)	12 (2.3)
Sex (Male)%	63.2	51.6	51.2
No formal education%	25.0	6.2	4.7
No formal occupation%	8.2	8.7	9.4
Radio at home%	69.5	88.8	98.4
Per capita monthly expenditure -$^1^	19.47	57.51	286.73
Household poor -%^2^	87.0	91.6	41.2
‘Preferred anti-malarial’:			
SP	43.9	57.5	45.3
Quinine	5.2	13.7	20.3
Halofantrine/Mefloquine	0	0	9.4
Chloroquine	1.4	2.5	6.2
Antipyretic/painkiller	4.9	5	1.6
Antibiotic	7.4	3.7	0
No preferred anti-malarial	36.8	17.5	15.6

One respondent (0.27%) residing in a rural area stated having a preferred anti-malarial but did not provide name.

^1^Exchange rate $1= Malawian Kwacha108.8374; average for February 2004; source: IMF (2004). Reflated to 2006 prices using CPI (All items – US Bureau of Labor Statistics; http://www.bls.gov/cpi/data.htm accessed 31 July 2007). n=28 cases had missing information on monthly expenditure; rural areas n=340 (6.60% non-response rate), in urban n= 63 (1.57% non-response), and peri-urban n= 77 (3.75% non-response).

^2^Defined as household expenditure per capita below poverty line. Poverty line in 2006: for urban areas, MK113.39 (US$ 0.91); rural south MK32.90 (US$0.25). The poverty line for urban areas was used to classify per capita expenditure in Peri-urban areas. Source: [21]http://www.ifpri.org/themes/mp18/malawipms/pmsprofile.pdf. Poverty lines reflated to 2006 using the Rural and Urban (all items) Consumer Price Indices for Malawi (NSO in http://www.nsomalawi.mw/component/content/article/21.html accessed 1 August 2007).

The reasons behind respondents declining to select any of the two drug profiles, was explored using logit regression analysis. Choosing none of the profiles was more likely without a formal occupation, but not related to any other of the respondent characteristics analysed: sex, education, bed net use, per capita household expenditure, and preferred anti-malarial (see Additional file 2). With regard to drug characteristics, the attribute levels of price and health professional recommendation were positively and negatively related to the probability of a scenario having no chosen profiles.

The (weighted) analysis of responses (rural n=2,782; peri-urban n=617; urban n=500; these numbers reflect the household population distribution in Zomba, and the over-sampling of peri-urban respondents from stratification, which is corrected with the weighted analysis) is presented in Table 2. This includes scenarios where indifference between the two options was stated (n=10 by 9 participants; 0.25% overall), in which case a random allocation to one of the two options was made. By increasing the random noise, this random allocation in effect reduces the magnitude of the estimated coefficients relative to the alternative of excluding those scenarios with indifferent options.

**Table 2:** **Discrete choice model estimates by model variant (Linear index - Equation **2)

	**Model 1: Normally distributed coefficients (N=508 respondents, 3899 responses)**		**Model 2: Log-normally distributed coefficients (N=508 respondents, 3899 responses)**	
**Variable**	**Estimate Coefficient [SD]**	**Standard error Coefficient [SD]**	**Estimate Coefficient [SD]**	**Standard error Coefficient [SD]**
Cost($)	−0.63 [0.52]	0.05*** [0.06]***	n/a	n/a
Fever duration^1^	−0.11 [0.03]	0.01*** [0.03]	n/a	n/a
Risk of rash	−1.27 [0.76]	0.09*** [0.10]***	n/a	n/a
Savings($) (i.e. inverse of cost)	n/a	n/a	−0.76 [1.59]	0.14*** [0.38]***
Days without fever (i.e. inverse of fever duration)	n/a	n/a	0.015 [0.02]	0.011 [0.02]
Probability of no side effect (i.e. inverse of risk of rash)	n/a	n/a	0.14 [0.90]	0.09 [0.13]***
Official treatment	3.06 [2.29]	0.24*** [0.22]***	3.44 [2.42]	0.32*** [0.32]***
Prophylaxis duration	0.05 [0.04]	0.004*** [0.005]***	0.06 [0.04]	0.005*** [0.006]***
Course duration	−0.29 [0.06]	0.05*** [0.11]	−0.32 [0.20]	0.06*** [0.13]
Relative variance: Urban vs. rural^1^	1.59 [0]	0.37	1.43 [0]	0.30
Relative variance: Peri-urban vs. rural^1^	0.91 [0]	0.19	0.85 [0]	0.19
−2 x(Mean simulated Log-likelihood ratio)^2^	47.4***		10.40	

Note: only coefficient estimates with asterisks alongside their standard errors had p<0.05.

*** p<0.001. n/a: not included in model.

1. Relevant test for relative variances is Ho: Variance urban/ Variance rural =1, and Variance peri-urban/ Variance rural=1, so p>0.05 (for urban vs. rural and peri-urban vs. rural, separately, and, or jointly using the simulated log-likelihood ratio tests –results not presented but available from authors).

2. Quasi-likelihood ratio test of the null hypothesis that all attribute fixed coefficients being equal to 0 (Note that in the case of Model 2, where three coefficients are log normally distributed –for cost, fever resolution, and risk of rash- this hypothesis is equivalent to their being jointly insignificantly different from 1, along with the normally distributed coefficients being zero). The test statistics are distributed in both cases as *χ*(6 degrees of freedom).

Model was estimated using simulation with n=100 repetitions.

Two models were estimated (see Table 2), Model 1 estimated normally distributed coefficients for all six attributes. An alternative model, Model 2, restricted the coefficients for cost, fever duration and risk of pruritus to the negative domain, in line with the logic that an individual would generally consider these as undesirable or ‘bad’ attributes, so that the higher their value, the less attractive the option would become. Model 2 can thus be considered as a form of sensitivity analysis to check the robustness of results obtained from Model 1. It is operationalized by specifying the parameters to be estimated in each case as the mean and standard deviation of the natural logarithm of the coefficient; since this restricts the original coefficients to be positive, the respective attributes (i.e. independent variables) are converted to negative values.

The direction of effect for all attributes was as expected. On the second column of Table 2, which shows results for the linear index in Equation 2, cost has a negative coefficient, implying that, in a given choice, the probability of selection of a drug would decrease (increase) as a result of an increase (decrease) in its price while all other attributes remain unchanged. Treatment duration has also a negative effect: drug therapies with longer treatment durations tend to be disregarded in favour of otherwise similar drugs; its coefficient magnitude (−0.29) relative to that for cost (−0.63) implies a marginal willingness to pay (accept) for shorter (longer) drug regimes equal to $0.46 per additional day without (with) treatment. For example, users would have to be compensated for suddenly having to use a treatment course that lasts 3 instead of 1 day by a price reduction of $0.92 if they were to remain as likely to use the new treatment as they were the old one (notice that at the lowest price experimented with, $0.46, that price reduction translates into a negative price so that users would have to be paid in order to entice them to entirely substitute the new, ‘ACT’, for the old, ‘SP’, option). A higher pruritus risk also discouraged selection of a drug treatment (p<0.001). Conversely, among two drugs that differ only in terms of prophylaxis duration, users would prefer, according to our findings, the one with the longer period of protection. Everything else equal, drugs with faster symptom relief were preferred, as were those recommended by a health professional.

In the effect of attributes other than speed of fever resolution and treatment duration, data are consistent with patient heterogeneity: a minority of individuals would value them contrary to the ‘common sense’ view. In the case of cost, a standard normal cumulative distribution of respondents with mean −0.63 and standard deviation 0.52 implies that 11.3% (z=1.21) of individuals had the view that, everything else being equal, more expensive drugs are more attractive. Similarly 4.7% (z=1.67) of individuals would find treatments with a higher risk of rash to be more attractive than less risky treatments. The SD coefficient for fever duration is not statistically different from zero at the 5% significance level, confirming the widely held association of speed of fever resolution with quality by anti-malarial drug users.

Results for Model 2 (fourth and fifth columns, Table 2) seem to confirm those for Model 1. The respective independent variables were adjusted to account for the fact that log-normal random coefficients are by definition positive, so that, for consistency with a priori expectations, these variables were entered in the estimation procedure as savings (negative costs) or goods (negative ‘bad’, i.e. for rash, the probability of no side effect, whilst for fever duration, the number of days without fever). The interpretation of the coefficients themselves is also different to that for Model 1 since a log normal distribution of mean zero is distributed around the value of 1; it may be seen that those coefficients corresponding to the probability of no side effects and days without fever, whose counterparts in Model 1 had values of −1.27 and −0.11, are not significantly different from zero at conventional levels and imply 95% CI of −1.38 to −0.96 and −1.03 to −0.99 under Model 2. Notice that the variance for days without fever does not appear significant at the 5% level. Other results are consistent with the results from Model 1.

Both models sought also to infer whether a difference existed between rural and other areas in the variance of the (logit model) error term. In Model 1, the estimated ratio of variances was 1.59 for the urban versus rural comparison, while that corresponding to peri-urban versus rural was 0.91. The test statistic for the relative variance coefficient of urban areas (Model 1: z= (1.59-1)/0.37 = 1.6) has an associated p=0.12, consistent with the variance ratio being 1 (equal variances) at the 5% level of significance. The respective test statistic for the peri-urban area (Model 1: z= (1–0.91)/0.19 = 0.48) has p= 0.63. Similar findings apply to Model 2. Therefore, the observed differences in variance between responses of individuals from different areas were found not to be statistically significant.

The analysis also explored the effect of covariates on stated choices and the robustness of random terms. The random coefficients of drug price, pruritus risk, prophylaxis effect duration and recommendation by a health professional were all robust to adding interaction terms between age, sex, lack of education, and monthly household expenditure covariates and the attributes (see Additional file 3). The effect of price was smaller in respondents from households with higher monthly expenditure, while it was larger among respondents with no education. After adjusting for these covariates, the interaction between the price attribute and an indicator for urban residence had p>0.05. The price and peri-urban interaction, however, was positive and had a p=0.032. Other interactions had p>0.05 (see Additional file 3).

Table 3 presents the sensitivity or elasticities of drug choice with respect to each attribute as a measure of their relative importance. The elasticity is the proportional change in probability of choosing an anti-malarial profile due to varying the value of one attribute divided by the proportional change in the value of the attribute (figures are absolute values). The elasticities are calculated with reference to the probability of choice for an official, one day treatment course, valued at $0.46, with 10 days of prophylactic effect and a 1 in 10 risk of rash developing from its intake, and that takes 12 hours to resolve the fever. The alternative drug profile is specified to be different only with respect to cost, at $1.10, and duration of treatment course, set at three days. At these values, the duration of fever is the most influential attribute (i.e. the one relative to which demand is most sensitive or elastic) whereas the duration of the prophylactic effect is the least important; a ten percent reduction in the speed of symptom (fever) resolution (i.e. from 12 to 13.2 hours) would result in a decrease in the likelihood that the affected drug therapy be chosen of 7.6% (i.e. from 77% to 71%); at the other extreme, a 10% increase in the duration of the prophylactic period (i.e. from 10 to 11 days) would only increase the likelihood of choice by 0.1%. Cost is seen as secondary to drug safety (i.e. the absence of rash), which in turn appears to be the second most important attribute. The duration of regimen is as influential as cost, and more so than duration of prophylactic effect of the drug. Health professional recommendation made drug selection more likely, by a median odds ratio of 36.4 (IQR: 2.1 -141.3).

**Table 3:** Elasticities of choice probability relative to each attribute*

**Attribute**	**Mean (SD)**	**Median (Interquartile range)**
Cost	0.08 (0.05)	0.06 (0.03 – 0.12)
Fever duration	0.76 (0.23)	0.88 (0.78 – 0.89)
Risk of rash	0.35 (0.29)	0.25 (0.08 – 0.62)
Duration of prophylaxis	0.01 (0.01)	0.02 (0.003 – 0.02)
Treatment course duration	0.08 (0.04)	0.09 (0.05 – 0.10)

*In absolute values. Based on results of estimated model 2, fixing variance to be the same across residential groups (urban, rural and peri-urban) and with a fixed coefficient for the treatment course duration and fever duration variables; the fixed part of the random (log normal) coefficient of the risk of rash variable was fixed at zero in view of estimation results (see columns 4 & 5 of Table 2).

Elasticities were calculated for an official treatment option with the following specification: Cost MK50 ($0.46), Time to Fever resolution 12 hours, Risk of Rash 1 in 10, Duration of Prophylactic effect 10 days, Treatment course duration 1 day. The alternative differed from the option whose attributes were varied in terms of Cost (MK120; $1.10) and Treatment Course Duration (3 days); the price is based on results of price-tracking retail surveys of ACTs subsidized by the Global Fund to Fight AIDS, TB and Malaria in Ghana, Kenya, Nigeria and Tanzania [22]. At these values the initial probability of use (market share) of the treatment option that is affected by the change of value in each attribute described by the Table is 76.9%. The predictions were calculated using n=50 repetitions.

The proportion of patients invariably choosing the option with the lower/higher attribute value or indicator (recommended by a health professional, ‘yes’/’no’) among those who happened to be allocated five or more scenarios with different values across options for the attribute in question was: cost, 17.2% (95% CI: 13 – 21); symptom resolution 4.4% (2 – 7); risk of rash, 26.3% (20 – 32); recommendation by health professional, 36.5% (30 – 43); prophylactic effect 21.8% (16 – 27); regimen duration 5.2% (2–8). That is, 63.5%, and possibly up to 70%, of individuals are willing to consider using drug treatments other than those endorsed by the formal healthcare system.

## Discussion

The results of this representative survey of heads of households randomly sampled from all households in the district of Zomba, Malawi, suggest that, on average, the five drug-related and the one healthcare policy tool attributes investigated significantly affect individual adults’ own treatment choices, and do so in the direction predicted a priori. There is heterogeneity in user appreciation of some attributes, i.e., cost, risk of side-effects (rash), treatment recommendation by a professional, and duration of prophylaxis. While for rash risk this implies counter-intuitive behaviour in a small minority of individuals that may result from misspecification of the normal functional form for random coefficients, for other parameters it may reflect genuine behavioural traits. For example, it is estimated that almost 9% of individuals would consider endorsement of an anti-malarial by a health professional as a disincentive to use it. This result is robust to accounting for household expenditure and level of education of respondents in the analysis.

Perhaps more significant is that up to 70% of individuals in Zomba may be prepared to consider using drugs other than those recommended by health professionals. This sheds light on the importance of official treatment policy for drug treatment choice in sub-Saharan Africa, suggesting that individuals in the community do not in general rely on the prescriptions of the formal system of those countries and that, to be effective, policy must be supported by other measures such as economic incentives from price subsidies [23]. This is in line with the findings of McCombie [1], and the observed frequency with which informal sources of health care are sought after fever onset in malaria endemic areas [16].

As expected, respondents were least willing to trade a higher rash risk for an improvement in other attribute: an estimated 20-32% of them would not do so. The most important attribute in drug selection was the speed with which an anti-malarial resolves fever; cost was only the third most important factor after pruritus risk and speed of fever resolution. In fact, the number of days that the course lasts for was found to be as important as cost. The prophylactic effect that drugs with slow rates of drug elimination may confer was of minimal influence on decisions to use an anti-malarial. These effects did not differ according to whether respondents lived in a rural, peri-urban or urban area.

Obtaining realistic choice scenarios is a common challenge in experimental studies. The frequent use of informal sources of treatment in the population [16] makes the experimental choice situation familiar and realistic enough for participants to provide valid responses to it. A further issue in experimental studies is the difficulty in conveying information on small risks or small variation of risks. The use of cartoon aids to communicate the notion of rash was intended to facilitate the response elicitation task to respondents, many of which would be illiterate, while avoiding practical and analytical problems associated with negative affective effects or fear induced by a more realistic graphic depiction of risk [24]. A relatively large variation in risk, from 1 to 3 in 10 cases, was selected and clearly accounted for by the participants’ responses. While responses from 4.7% of individuals appear to imply, paradoxically, that high risk treatments are preferable to otherwise similar treatments of low risk, this result was not mediated by lack of education, which suggests that illiteracy was not a barrier to respondents’ understanding the task and may reflect the benefits of using cartoon aids. The experimental design allowed for indifferent responses and these turned out to represent a negligible proportion of the total. It may be relevant to assess smaller risk levels and differences, and using cartoon aids as in this study represents a promising approach to the task.

The results highlight the ongoing challenges to acceptance of artemisinin combination therapy (ACT), which is more expensive and requires a longer treatment course than SP, the former official first-line treatment still widely used in the region and still available in some informal outlets. A price subsidy on its own is likely to have limited effect: bringing the price down to the same level as that of SP would result in a predicted likelihood of choice for ACT of 63% given a scenario with SP as the alternative. In order to compensate for the disadvantage of using ACT in terms of course duration (3 vs. 1 day), co-formulating the drug with or ensuring co-prescription of an antipyretic to shorten the time to perceived symptom resolution by users may be an effective option to increase use; indeed, there is scope to pay for such compounded treatment through the implicit willingness of respondents to trade off a lower price subsidy for faster symptom control.

In terms of social welfare, a non-zero price coefficient of demand for an essential commodity to public health, such as anti-malarial drug therapy in sub-Saharan Africa, suggests its susceptibility to monetary incentives independent of need and, consequently, possible misuse (i.e. ‘moral hazard’ in consumption). The same (negative) price and attribute coefficients were estimated to apply to rural, urban, and possibly peri-urban areas, implying that willingness to pay for anti-malarial drug attributes was the same across them. While the size of the study may have been insufficient to estimate differences across locations precisely, given that the cost of living in urban areas of southern Malawi is almost four times that in rural ones, the same nominal estimate of willingness to pay for an attribute in both areas implies a higher economic value to rural residents. This may partly reflect implicit assessments of differential ease of access to providers between urban and other areas, since physical access was not explicitly controlled for in the experiment. It seems remarkable that nominal willingness to pay is the same across areas under the observed disparities between the urban, rural and peri-urban samples in terms of socio-economic living conditions, education and household income. Thus while the effectiveness of monetary incentives, such as price subsidies for ACT, is likely to be the same across the three subpopulations, optimization of social welfare under limited resources may require allocating a higher drug price subsidy to rural than to urban areas.

## Conclusions

In summary, this study found that use of cartoon aids to convey magnitudes of risk and other clinical attributes facilitates the implementation of discrete choice experiments in populations with high levels of illiteracy. Head of households in a malaria endemic area of sub-Saharan Africa are willing to use antimalarial treatments that while conveniently addressing their symptoms may not be those endorsed by health professionals. The results suggest that policies aiming to improve the treatment of malaria cases in the community should consider the provision of economic incentives to users of a new anti-malarial as a limited part of effective policy implementation measures. Indeed, given the limited margin for price reductions, a full price subsidy complemented by a conditional cash or in-kind transfer policy may be required to steer users away from ineffective treatments and into new effective ones. The study highlights the importance of addressing the disaffection with the healthcare system in sub-Saharan Africa, which predisposes individuals to consume suboptimal treatments for potentially life threatening endemic conditions [1,25]. Further studies should seek to conduct discrete choice experiments with adequate power to address the question of the magnitude of differences in price and treatment attribute sensitivity between anti-malarial drug users of rural and urban areas in order to determine optimal subsidies.

## Competing interests

The authors declare that they have no competing interests.

## Authors’ contributions

AML conceived the study; all authors participated in the study design. AML coordinated the whole study; EDKL coordinated the data collection in Malawi; AML prepared the data for analysis; REMM conducted the statistical analysis and prepared the first draft of the manuscript; AML and DGL contributed in drafting the manuscript. All authors read and approved the final manuscript.

## Supplementary Material

Additional file 1 **Attributes and levels.** Description: This word file provides the attributes and levels using the cartoon aids. Click here for file

Additional file 2 **Statistical analysis of the probability of choosing none of the drug profiles in a choice question.** Description: The data provided are the results of statistical analysis of explanatory factors of the probability of not choosing any of the two options in a scenario. Click here for file

Additional file 3 **Sensitivity analysis of random effects of attributes on treatment choice.** Description: The data provided are the results of extending the statistical analysis (Model 1) to account for the possible effect of interactions between respondent covariates and drug treatment attributes on the probability of choosing a treatment. Click here for file
