# Supplementary figures and images for: Stated preferences for anti-malarial drug characteristics in Zomba, a malaria endemic area of Malawi
Source: Malar J. 2014 Jul 8;13:259. doi: 10.1186/1475-2875-13-259 (PMC4108233; doi:10.1186/1475-2875-13-259)

**Additional file 1. Attributes and Levels**


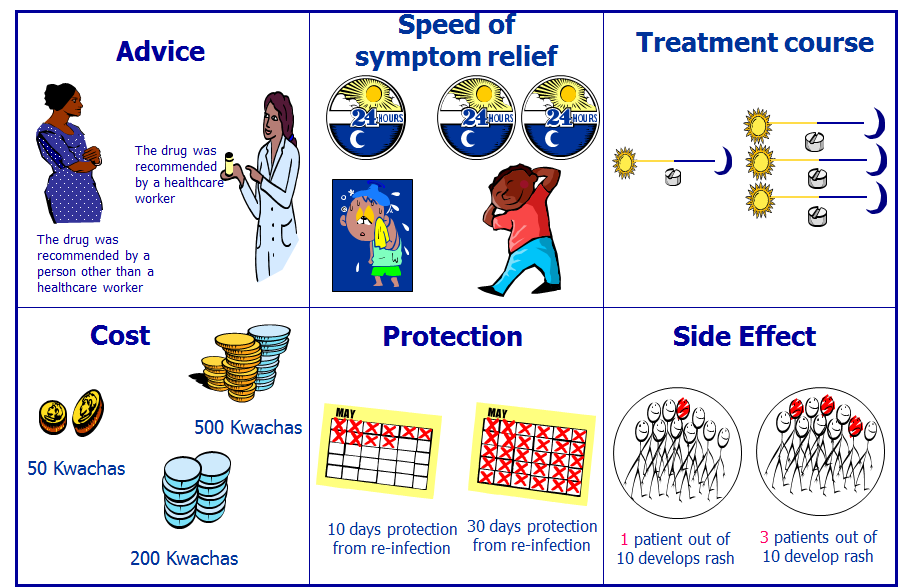

Supplement: Additional file 1 — Attributes and levels. Description: This word file provides the attributes and levels using the cartoon aids. [file 1475-2875-13-259-S1.docx]
