# Supplementary material for: Stated preferences for anti-malarial drug characteristics in Zomba, a malaria endemic area of Malawi
Source: Malar J. 2014 Jul 8;13:259. doi: 10.1186/1475-2875-13-259 (PMC4108233; doi:10.1186/1475-2875-13-259)
Supplement: Additional file 2 — Statistical analysis of the probability of choosing none of the drug profiles in a choice question. Description: The data provided are the results of statistical analysis of explanatory factors of the probability of not choosing any of the two options in a scenario. [file 1475-2875-13-259-S2.docx]

**Additional file 2. Statistical analysis of the probability of choosing none of the drug profiles in a choice question**

Thirteen percent of respondents did not provide a drug choice to at least one of their questions, resulting in 4.04% choice questions with no chosen drug profile. The table below presents the results of a probit regression analysis of factors influencing the probability of obtaining a drug profile in response to a choice question. The analysis accounts for non-independence of response probabilities due to the multiplicity of scenarios presented to the same individual; the estimated random variance across respondents is however not significantly different at conventional levels (p>0.05), consistent with no conditional (unobserved) respondent heterogeneity. Those without a formal occupation were more likely to make a choice. None of the other socio-economic variables appears to influence the probability of selection nor were those who preferred SP (the current first line), or Quinine Mefloquine or Halofantrine treatment more or less likely to select one of the two hypothetical treatment options than other individuals (i.e. those without a ‘preferred anti-malarial treatment’ or who identified as such a brand name corresponding to an antipyretic, painkiller, antibiotic or Chloroquine). Further, those participants reporting bednet use the previous night did not differ in their probability of selecting one of the two hypothetical drug options from other individuals. As for drug characteristics, the maximum value across the two options for an attribute considered as ‘good’ a priori (i.e. prophylactic effect, health professional recommendation) and the minimum value across options of a ‘bad’ attribute (i.e. price, pruritus risk, time to symptom resolution and regimen duration), was included as covariate for the respective attribute and scenario. The minimum available price and availability of an option with a health professional recommendation had a negative and positive effect on the probability of response, respectively. Scenarios in which the option with the faster symptom resolution takes longer to produce the effect appear to be less likely to result in a choice, although the estimate does not reach conventional significance. Other attributes also had a p>0.05.

**Table I Probability of selecting a drug profile (hierarchical probit regression)**

|  | **Sample with complete data**  **N=480 respondents, 3839 choice questions** | |
| --- | --- | --- |
|  | **Odds Ratio** | **95% CI** |
| **Minimum cost of the two options**  (per $1 increase) | 0.37 | 0.24- 0.56** |
| **Minimum time to fever resolution of the two options**  (per hour increase) | 0.97 | 0.92- 1.02 |
| **Minimum treatment duration of the two options**  (per extra day of treatment) | 0.87 | 0.63- 1.20 |
| **Minimum rash risk of the two options**  (per extra case in 10 individuals) | 0.96 | 0.70- 1.31 |
| **Longest prophylactic effect of the two options**  (per extra day of protection) | 1.00 | 0.98-1.02 |
| **At least one option was recommended by health professional**  (yes vs. no) | 18.08 | 7.91- 41.34 ** |
| **SP preferred treatment^1^** | 1.44 | 0.76- 2.73 |
| **Other preferred anti-malarials^2^** | 1.64 | 0.53- 5.09 |
| **Sex** | 1.01 | 0.58- 1.76 |
| **Bednet use previous night** | 0.67 | 0.33- 1.38 |
| **No education** | 1.09 | 0.51- 2.34 |
| **No formal occupation** | 5.23 | 1.04-26.38* |
| **Household expenditure ($ per capita)** | 1.00 | 0.99- 1.02 |
| **Unexplained respondent Heterogeneity (variance)** | 34.56 | 22.37 |

^1^ Indicator of cases stating one of a number of brand names of sp to identify their preferred anti-malarial treatment.

^2^ Indicator of cases stating the brand names of Quinine, Mefloquine or Halofantrine to identify their preferred anti-malarial treatment.

* p<0.05; **p<0.001.
