# Supplementary material for: Stated preferences for anti-malarial drug characteristics in Zomba, a malaria endemic area of Malawi
Source: Malar J. 2014 Jul 8;13:259. doi: 10.1186/1475-2875-13-259 (PMC4108233; doi:10.1186/1475-2875-13-259)
Supplement: Additional file 3 — Sensitivity analysis of random effects of attributes on treatment choice. Description: The data provided are the results of extending the statistical analysis (Model 1) to account for the possible effect of interactions between respondent covariates and drug treatment attributes on the probability of choosing a treatment. [file 1475-2875-13-259-S3.docx]

**Additional file 3. Sensitivity analysis of random effects of attributes on treatment choice**

**Table Discrete choice models with covariate interactions (Linear index - Equation 2)**

|  | **Mixed logit model with normally distributed coefficients (Variants of Model 1)** | | | |
| --- | --- | --- | --- | --- |
|  | N respondents= 479, N responses= 3687 | | | |
|  | Attributes only  (A) | A + cost by urban/peri-urban interactions  (B) | B + cost by socio-economic interactions (C) | Covariate interactions with all attributes  (D) |
| Variable | Estimate  Coefficient  [SD] | Estimate  Coefficient  [SD] | Estimate  Coefficient  [SD] | Estimate  Coefficient  [SD] |
| Cost($) | -0.75***  [0.56] | -0.75***  [0.52]*** | -0.69***  [0.51]*** | -0.58***  [0.55] |
| Fever duration | -0.12***  [0.02] | -0.12***  [0.03] | -0.12***  [0.01] | -0.12***  [0.02] |
| Risk of rash | -1.47***  [0.92] | -1.43***  [0.84]*** | -1.42***  [0.83]*** | -1.68***  [0.90] |
| Official treatment | 3.47***  [2.56] | 3.31***  [2.39]*** | 3.32***  [2.40]*** | 3.59***  [2.46] |
| Prophylaxis duration | 0.06***  [0.04] | 0.06***  [0.04]*** | 0.06***  [0.04] | 0.05***  [0.04] |
| Course duration | -0.32***  [0.00] | -0.32***  [0.00] | -0.32***  [0.00] | -0.33***  [0.12] |
| Cost x monthly expenditure /100 |  |  | 0.03 | 0.02 |
| Cost x age |  |  |  | -0.05 |
| Cost x gender |  |  |  | 0.06 |
| Cost x no education |  |  | -0.30* | -0.25* |
| Risk of rash x age |  |  |  | 0.02 |
| Risk of rash x gender |  |  |  | 0.22 |
| Risk of rash x no education |  |  |  | 0.22 |
| Official treatment x age |  |  |  | -0.07 |
| Official treatment x gender |  |  |  | 0.31 |
| Official treatment x no education |  |  |  | 0.41 |
| Prophylaxis x age |  |  |  | 0.00 |
| Prophylaxis duration x gender |  |  |  | 0.00 |
| Prophylaxis duration x no education |  |  |  | -0.01 |
| Cost x urban |  | 0.20* | 0.07 | 0.12 |
| Cost x peri-urban |  | 0.42* | 0.36* | 0.42* |
| -2*(Mean simulated Log-likelihood ratio) | A vs fixed effect  χ(6)=49.30*** | B vs A χ(2)=9.77* | C vs B  χ(2)=8.86* | D vs. C  χ(11)=16.95 |

x Denotes interactions; * p<0.05; p**<0.01; ***p<0.001; χ(#) denotes chi-squared statistic with # degrees of freedom. Other statistical tests used the ratio of the estimate and its standard error as an asymptotically standard normally distributed statistic (figures in brackets are standard deviations and cannot take negative values; although applying standard normal critical values to their ratio statistics leads to biased inferences, it provides a reliable approximation given the magnitude of the test statistics in the present case). Bottom row presents likelihood ratio tests of nested models (first column is variant A vs A including only fixed effects of attributes, i.e. without the random component).

The table presents the results of estimating variants of Model 1: In Model A, the heterogeneity (scale) parameters are fixed at the value of 1, so that the error term is homoscedastic (unlike Model 1, which allowed for the residual variance to differ between rural, peri-urban and urban areas). Model B adds two interaction covariates for the cost variable, one for the urban indicator and another for the peri-urban one. Model C includes, in addition to the regressors included in Model B, two interaction terms for the cost attribute; one, with an indicator taking the value of 1 if the person reported no formal education, and 0 otherwise; the other is an interaction with the level of monthly household expenditure. Model D includes the regressors of Model C and interaction terms for each of the four attributes with a statistically significant (p<0.05) standard deviation in Model 1 (i.e. cost, risk of rash, prophylaxis duration and recommendation by health professional –i.e. official treatment) with age, gender and the indicator for lack of education. An interaction term with monthly expenditure was not included for attributes other than cost, because of no obvious a priori rationale for any such interaction having an effect on choice.

Results for Models A-D suggest that the statistically significant random variability found for the effects of cost, risk of rash, official treatment (recommendation by health professional) and prophylaxis duration attributes in Model 1 is not explained by age, sex or the level education. In Model B, the positive coefficient estimated for the cost by urban and cost by peri-urban interaction terms indicate that the negative effect of a drug’s price on the probability of its being chosen is lower in urban and peri-urban areas than in rural areas. Model C results suggest that the lower influence of price (cost) on drug choice among urban residents relative to rural ones is explained by differences in the prevalence of lack of formal education across the two groups, and possibly by differences in economic welfare as approximated by monthly household expenditure (although the effect of this interaction has a p=0.17). Residents of peri-urban areas, however, do appear to be less susceptible to price considerations in their choice of drug treatment than rural residents are (p=0.03), even after controlling for lack of education and level of household expenditure. Other covariates seem to have limited explanatory power as evidenced by a quasi-likelihood ratio test that is not statistically significant by conventional standards (last row, last column, Model D χ(11)=16.95, p>0.10).
